# Docetaxel Rechallenge in a Heavily Pretreated Patient With Castration-Resistant Prostate Cancer: A Case Report and Review of Literature

**DOI:** 10.1097/MD.0000000000002754

**Published:** 2016-04-08

**Authors:** Giuseppe Di Lorenzo, Martina Pagliuca, Teresa Perillo, Alfonso Benincasa, Davide Bosso, Sabino De Placido, Carlo Buonerba

**Affiliations:** From the Medical Oncology Unit, Department of Clinical Medicine, Federico II University, Naples, Italy.

## Abstract

Chemotherapy agents for patients with metastatic castration-resistant prostate cancer (mCRPC) include docetaxel and cabazitaxel. Although docetaxel is approved in the first-line treatment setting, a few studies have shown that selected patients can benefit from docetaxel rechallenge.

We, here, report the case of a heavily pretreated mCRPC patient who reported clinical benefit from receiving docetaxel after previous exposure to docetaxel, cabazitaxel, abiraterone, and enzalutamide.

After 4 cycles of treatment, patient's performance status had improved to 1, the hemoglobin level was 12.9 g/dL and his serum prostate specific antigen levels were reduced by >70%, with no treatment-related adverse events.

Although docetaxel rechallenge is a therapeutic option for selected patients, the risk of cumulative toxicity described in literature must be carefully considered.

As the risk of cabazitaxel-related cumulative toxicity is probably lower, retreatment with cabazitaxel rather than docetaxel may also be an option in the setting of heavily pretreated mCRPC patients.

## INTRODUCTION

Although the incidence of metastatic prostate cancer has dropped after the introduction of prostate specific antigen (PSA)-based screening,^1^ prostate cancer remains a major cause of cancer-related death in men, with 27,540 men estimated to die of the disease in the US in 2015.^2^ Docetaxel was the first systemic agent to be associated with a clinically and statistically significant improvement in the life expectancy of men with metastatic castration-resistant prostate cancer (mCRPC),^3^ and an even greater benefit is expected to be associated with its combined and sequential use with several recently approved treatments.^4,5^ Novel agents approved in mCRPC include chemotherapy (cabazitaxel), hormonal (abiraterone, enzalutamide), immunotherapy (Sipuleucel-T), and radiopharmaceutical (radium 223) agents.^4,5^ Despite a wealth of novel therapies approved in the post-docetaxel setting, some patients may still be candidates for retreatment with docetaxel,^6–8^ although the risk of cumulative toxicities associated with docetaxel must be carefully considered.^9^ We, here, report the case of a heavily pretreated mCRPC patient who was re-treated with docetaxel after previous exposure to docetaxel, cabazitaxel, abiraterone, and enzalutamide. Docetaxel rechallenge was associated with a >70% PSA levels decrease, an improvement in performance status, a reduction of pain levels and need of opioids, a resolution of anemia, and no remarkable treatment-related adverse events, as described in detail below.

### Case Report

The patient, a man, born in 1935 presenting with no comorbidities other than essential hypertension, had been diagnosed with metastatic prostate cancer at a different Institution in 2005 (Gleason 3+3 on biopsy, multiple bone metastases on bone scan, no lymphnode or parenchymal metastases on computed tomography (CT) scan, PSA = 45 ng/mL). The patient came to our attention because of progressive disease in November 2011, after being treated with continuous androgen deprivation therapy and receiving 12 cycles of 3-weekly docetaxel (75 mg/m^2^) with last administered cycle in April 2011. Serum PSA levels were 288 ng/mL, while a whole body CT scan with and without contrast showed enlarged lymph node metastases in the Barety space (shortest axis, 20 mm) and multiple bone metastases were detected on bone scan (ribs, vertebrae, long bones, and facial skeleton). His Eastern Cooperative Oncology Group (ECOG) performance status was 0 and his pain was well controlled with opioids (visual analog scale [VAS] = 2/10; oxycodone, 20 mg/day). The patient received 14 cycles of cabazitaxel (25 mg/m^2^, 3-weekly) plus oral prednisone (5 mg/day) and zoledronic acid (4 mg, 3-weekly) from November 2011 (7 months after prior docetaxel) to August, 2012.

In September, 2012, CT scan showed shrinkage of the mediastinal lymph-nodes (shortest axis, <1 cm), while bone scan showed reduced tracer uptake of the pre-existing lesions. Serum PSA levels had dropped to 14.6 ng/mL. Adverse events had been consistent with cabazitaxel safety profile and included G2 anemia, G2 leukopenia, G4 neutropenia, and G2 asthenia. The patient continued to be treated with androgen deprivation therapy plus zoledronic acid until July 2013, when bone scan showed bone progression, with poorly controlled osseous pain despite active pain management (VAS = 7/10; oxycodone, 60 mg/day; 8 Gy external beam radiation therapy to the femoral head). The performance status of the patient was 2, and his serum PSA level was 186 ng/mL. The patient subsequently received 15 cycles of abiraterone acetate (1000 mg/day for 30 days) plus oral prednisone (10 mg/day) until November, 2014. Abiraterone was associated with improvement in pain and in performance status, and the main treatment-related adverse events included G2 asthenia and G1 anemia.

Disease progression was reported in November, 2014, when widespread osseous metastases in the femurs, ribs, vertebrae, and pelvis were detected on bone scan, while a whole body CT scan with and without contrast revealed multiple enlarged lymph nodes metastases in the subcarenal region and in the Barety space (maximal shortest axis, 4 cm). The PSA level was 474 ng/mL. Further, radiological progression was reported in March, 2015 after a few months’ treatment with enzalutamide (160 mg/day). In June 2015, PSA levels were ≈1500 ng/mL and hemoglobin levels were 7.7 g/dL. Pain could not be adequately controlled with opioids (oxycodone, 80 mg/day; VAS = 9/10). Patient's performance status was 2. In an attempt to provide symptomatic palliation, the patient was rechallenged with weekly docetaxel, which was administered on days 1, 8, 15 cycled every 28 days at 30 mg/m^2^ along with darbopoetin (150 mcg weekly). After 6 cycles of treatment, patient's performance status had improved to 1, the hemoglobin level was 12.9 g/dL and his serum PSA levels were reduced by >70%, with no treatment-related adverse events (Table 1). As of December 20th, treatment with docetaxel is still ongoing.

**TABLE 1:**
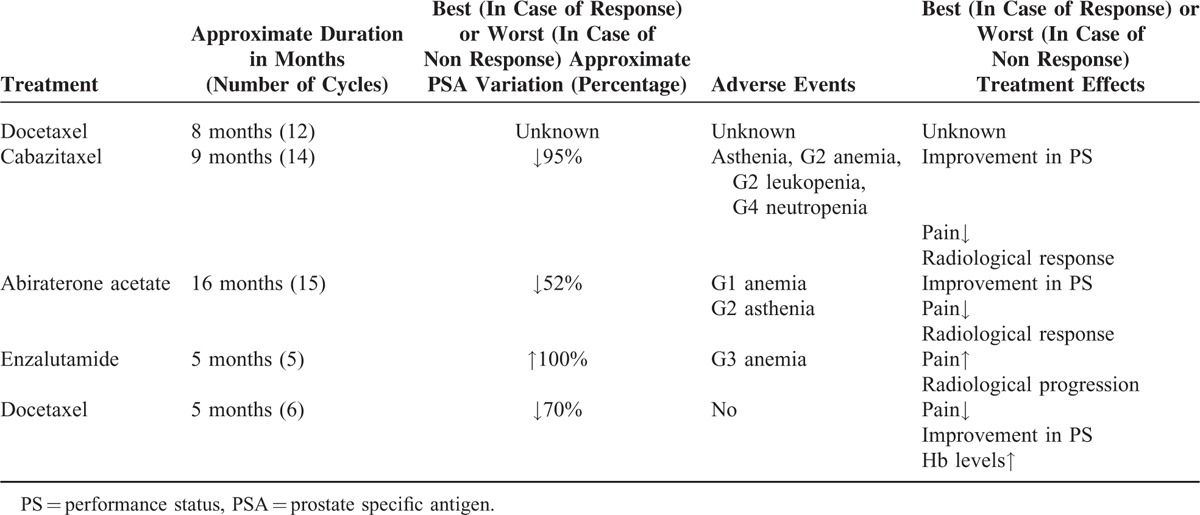
History of Patient's Treatment

## DISCUSSION

Docetaxel was the first systemic agent to show effectiveness in mCRPC. In the 1006 patients with mCRPC enrolled in the phase III trial by Tannock et al,^10^ docetaxel (vs. mitoxantrone) administered every 3 weeks was associated with a significant hazard ratio for death of 0.76. Furthermore, approximately one third (vs. one fifth) of patients treated with either 3-weekly or weekly docetaxel (vs. mitoxantrone) experienced a significant decrease of pain levels. Of note, while weekly docetaxel was not associated with a survival improvement versus mitoxantrone, 3-weekly (vs. weekly) docetaxel was associated with an increased rate of grade 3 to 4 neutropenia (32% vs. 2%), which is the reason why we chose to administer weekly (rather than 3-weekly) docetaxel in our elderly, heavily pretreated patient with impaired bone marrow function.^11^ Both 3-weekly and weekly docetaxel was associated with a very low incidence (<5%) of grade 3 to 4 anemia,^10^ so the potential benefits on bone marrow function of docetaxel-induced tumor regression outweighed the risk of severe treatment-induced anemia in our patient with grade 3 anemia at baseline. Although retreatment with docetaxel has been associated with PSA responses and clinical benefit both in prospective^7,8^ and retrospective studies,^9^ its effectiveness has not been assessed in a phase III trial.^6^ In the phase II trial by Di Lorenzo et al,^7^ 45 patients with a prior response to docetaxel and a biochemical relapse free survival of at least 5 months since docetaxel suspension received a median of 3-weekly docetaxel cycles at standard doses. A >50% PSA reduction was reported in 24.5% of patients, with a median progression-free survival (PFS) of 5 months and a median overall survival (OS) of 13 months. Grade 1, 2, 3, and 4 peripheral neuropathy were, respectively, reported in 13.3%, 4.4%, and no patients.

Caffo et al^8^ assessed all the variables potentially capable of predicting the response to docetaxel rechallenge. 46 patients underwent 92 rechallenges. The overall biochemical response rate was 66%. Multivariate analysis showed the response to the previous cycle and the time from the previous cycle were predictive of the response to a rechallenge.

In a retrospective study by Oudard et al including 270 men who had previously benefited from first-line docetaxel,^9^ no difference was reported between the OS of the 223 patients who were rechallenged with docetaxel and the OS of the 47 men who received non-taxane-based therapy, but improved PSA response and symptom relief were associated with docetaxel rechallenge. Data about the efficacy and safety of docetaxel rechallenge in the setting of patients pretreated with novel hormonal and chemotherapy agents are lacking. The lack of efficacy of enzalutamide reported in our case after exposure to abiraterone and docetaxel is consistent with previously reported findings,^12^ and may be associated with the selection of neoplastic clones expressing the androgen-receptor splice variant 7 (Androgen Receptor [AR]-V7),^13^ which causes resistance to enzalutamide and abiraterone.^11^ Conversely, AR-V7 is not associated with resistance to taxanes,^14^ which is consistent with the potential effectiveness of docetaxel rechallenge in patients refractory to novel hormonal agents. While docetaxel rechallenge is clearly not feasible in men with primary refractoriness to docetaxel, which is likely to be a negative prognostic factor,^15^ its use in clinical practice may be increasingly frequent due to the results obtained with docetaxel in castration-sensitive prostate cancer patients with metastatic disease,^16^ and especially in patients with a high Gleason score, who may derive the greatest benefit from taxane-based chemotherapy.^17,18^ Nevertheless, cumulative toxicity associated with docetaxel must be carefully considered. In the retrospective study by Caffo et al,^8^ grade 3 to 4 nail disorders and sensory neuropathy were reported in 4.6% and 9.2% of the 87 patients receiving “first” docetaxel rechallenge and in 7.9% and 7.9% of the 38 patients receiving “second” docetaxel rechallenge. These adverse events are expected to be significantly less common with cabazitaxel.^19^ Furthermore, a retrospective analysis of patients enrolled in the Italian Expanded Access program on cabazitaxel did not identify any sign of cumulative toxicity.^20^

Additional therapeutic options have also been considered as an alternative to docetaxel rechallenge in the case presented here. These included radiopharmaceutical agents^21^ and platinum-based chemotherapy.^22,23^ While radium 223 is not yet available at our Institution, strontium-89 and samarium-153 were not considered a viable option because of the limited survival expectancy estimated of the time of docetaxel rechallenge, and also because both of these agents were associated with a grade 3 to 4 anemia in approximately 10% of cases.^20^ Platinum-based chemotherapy was also excluded because of concerns of toxicity. In a preliminary report by Buonerba et al^23^ of 15 heavily pretreated mCRPC patients receiving carboplatin plus etoposide, grade 3 to 4 anemia was reported in 4 patients (≈25%), while the patient was clearly ineligible to cisplatin treatment (poor performance status, older age, and grade 3 anemia).

## CONCLUSIONS

In conclusion, we reported the first case of a patient treated with docetaxel rechallenge after receiving salvage treatment with cabazitaxel, abiraterone, and enzalutamide. A clinical benefit was achieved along with minimal toxicity. Due to the risk of docetaxel-related cumulative toxicity, retreatment with cabazitaxel may also be a feasible option in the setting of heavily pretreated patients.
